# Exploring Destination Choice Intention by Using the Tourism Photographic: From the Perspectives of Visual Esthetic Processing

**DOI:** 10.3389/fpsyg.2021.713739

**Published:** 2021-11-18

**Authors:** Weiwei Deng, Yingxing Lin, Lijun Chen

**Affiliations:** ^1^School of Economics and Management, Fuzhou University, Fuzhou, China; ^2^Aesthetic Education Research Center, Fuzhou University, Fuzhou, China; ^3^School of Humanities and Social Sciences, Fuzhou University, Fuzhou, China

**Keywords:** environment esthetics, photograph esthetics, visual esthetics, first impression, esthetic emotion, visual appeal, choice intention, eye-tracking

## Abstract

This study aims to deepen the understanding of tourism photography by developing and testing a theoretical model that accounts for the relationships between visual esthetics and destination choice intention of tourists. Thus, we sought to use a stimulus-organism-response (S-O-R) paradigm to predict destination choice intention, which includes three variables related to visual esthetics: first impression, visual appeal, and esthetic emotion. We used the combination of self-reported and eye movement data to examine the cognitive processes of tourists that visual esthetic formation. We found that compared to the built environment and amateur esthetic images, natural environment and professional esthetic images can get (1) higher visual appeal, (2) better first impression, and (3) higher visual processing fluency (or less cognitive effort) and positive esthetic emotions. Furthermore, visual appeal, first impression, and esthetic emotion deriving from environment esthetics and photograph esthetics have a positive impact on destination choice intention. This study has practical implications for destination planning and management.

## Introduction

In the visual information age, vision is the most direct and fastest sensory channel for an individual to receive information. Many feelings are affected by the initial visual perception. From the perspectives of the tourists, a photographic image as one of the most salient visual information can unconsciously influence their choice behaviors (Ngan et al., 2020). From the perspectives of the destination providers, the photographic image is crucial for managers owing to its relationship with destination marketing strategies. Tourism is an invisible product that requires several pictures (Rakic and Chambers, 2010). Tourism images can present the image of the destination, attract the attention of tourists, and affect their perception and reactivity (Li et al., 2016). Various tourist platforms provide a large number of pictures, which can affect the choice of a tourist destination (Iordanova and Stainton, 2019), satisfaction (Ramseook-Munhurrun et al., 2015), loyalty (Prayag et al., 2017), willingness to revisit (Assaker et al., 2011), and behavioral intention (Wang and Sparks, 2016). Therefore, tourist photos have become an important reference source for tourists to perceive the image of destination and travel choice (Ma et al., 2018), which has aroused widespread interest in academic tourism circles. However, the destination image is an important visual representation of a tourist destination, and the research on image information is rough and lacks fully studied (Balomenou and Garrod, 2014). Research on the behavior of the destination image perception, many scholars focus on the differences in individual culture and regional perception, tourist destination perceived attraction, the components of destination image perception, etc. (Balomenou and Garrod, 2019). Few scholars use destination images as visual perceptible materials to study the influence of destination choice intention from the perspective of visual esthetics (Hao et al., 2015).

In psychology, the first impression is crucial for how the user perceives and acts, such as trust, revisit, and purchase behavior (Król and Zdonek, 2020). Lindgaard et al. (2006) found that people can evaluate the website esthetics in 50 ms. Their findings suggest that esthetics can be developed in the context of the first impression of users. In website interfaces, the esthetic is a key factor that users inference outer appearance (Iten et al., 2018). In addition, the study of esthetics is important to the tourism industry, and beauty is considered an important feature of the tourist destination and experience (Knudsen et al., 2015). A recent study of visual appeal revealed that a good destination image can attract many tourists and even affect their willingness to revisit (Assaker et al., 2011). The beauty of travel photos is based on the pleasant emotional response of an individual to the visual scene. It has long been considered that the destination which has enjoyed a high visual appeal can positively influence the decisions of tourists (MacKay and Couldwell, 2014). The esthetic is developed when subjective is influenced by these feelings, and the emotion of an individual may influence a potential preference (Xenakis and Arnellos, 2014). However, few studies have explored the connection of visual esthetic with the destination choice intention of tourists. According to the theories of esthetics, the first stage of esthetic is generating a global impression at the first glance, where the first impression includes early affective. In addition, if the information on the photo has sufficient interest, the second phase of esthetic often ensue, which is cognitive processing (Locher, 2015). Thus, we sought to use a stimulus-organism-response (S-O-R) paradigm to predict destination choice intention, which includes three variables related to visual esthetics as follows: first impression, visual appeal, and esthetic emotion, and aim to explore the connection of visual esthetics with a destination choice intention of tourists.

## Theoretical Background

### Stimulus-Organism-Response Paradigm

The S-O-R framework is based on environmental psychology to explain the impact of environmental stimuli on human behavior (Kirillova and Chan, 2018). S is the environment that an individual encounter at a given moment. O components are described as “prior experiences, knowledge, beliefs, motivations, personal personalities, feelings, etc.” Customer R is basically the desire to enter or leave a particular environment. From the perspective of consumer psychology, environmental stimuli affect the cognitive and emotional processing of consumers, thereby affecting their behavior. The cognitive state involves the process of obtaining and interpreting individual information, while the emotional state is the process of emotional response to environmental stimuli. Knudsen et al. (2015) considered that the S-O-R framework is a more suitable model to study visual appeal and found that the design esthetics of the application can influence the adoption and recommendation intention of an individual.

### Visual Esthetic

Thomas Aquinas claimed that beauty is commonly applied to things that are pleasing either to understanding, imagination, or the senses of an individual (Lavie and Tractinsky, 2004). The general esthetic interpretation of beauty is related to perception and delight. Esthetic pleasure is a natural response in humans. Therefore, individual differences lead to different degrees of esthetic pleasure. Baumgarten first defined esthetics as the science of the senses, or the cognition obtained from the processing senses of an individual (Dickie, 1997). Esthetics refers to the feelings of individuals toward an object and indicates the correspondence between those feelings and taste or purpose of individuals (Thuering and Mahike, 2007).

Visual esthetics is related to the preference of a person that represents their sense of beauty toward images, which can influence perceptions, the trustworthiness of a website, and critically affect user satisfaction and pleasure. Visual esthetic identification can help deeply understand human preference and online social behavior (Moshagen and Thielsch, 2010; Bari et al., 2020). In tourism, esthetics and tourism are connected philosophically; in fact, esthetic experience is an essential element of tourism. Breiby (2014) explored esthetic dimensions in a nature-based tourism context which included perception, structure, senses, beauty, and pleasure. Natural esthetics involve interactions between visitors and nature. A natural esthetic experience is formed by the unidirectional construction of individuals regarding nature. This experience highly depends on tourists, whose perceptions of nature are influenced by their existing cultural backgrounds.

### Photography Esthetics and Environmental Esthetics

On the one hand, photographic images about destinations on online platforms may depict professional or amateur by photograph esthetics. Professional esthetic photos are usually produced by destination managers, while amateur esthetic photos are usually produced by tourists. Prior study shows that photos with professional esthetics have esthetically pleasing content and should yield more positive responses, while photography esthetics can affect the visual appeal of the destination and tourist booking intention (Marder et al., 2021). On the other hand, photographic images may depict natural or built environments by the environment photograph type (van den Berg et al., 2003). In the marketing images of the hotel, Wang et al. (2018) considered that nature-based servicescape can attract the visual attention of customers and stimulate behavior intention of consumers more than built-based servicescape. The beauty of the natural environment has a strong appeal, and it can evoke individuals to relax and enjoy being close to.

### Research Framework

**FIGURE 1:**
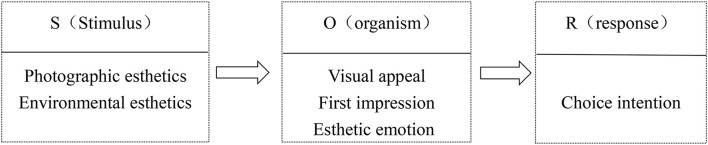
Research framework based on the stimulus-organism-response (S-O-R) framework.

Taken together, combined, S-O-R framework, visual esthetics, environmental esthetics, and photographic esthetics can provide the research framework of this study, as shown in Figure 1. Among them, we took photography esthetics and environmental esthetics of online travel pictures as S factors. Second, according to theories of esthetics, the first stage of esthetic is generating a global impression with the first glance, where the first impression includes initial affective. In addition, if the information on the photo has sufficient interest, the second phase of esthetic often ensue, which is cognitive processing (Locher, 2015). Therefore, we regarded visual esthetics of tourists as an O component, and first impression, visual appeal, and esthetic emotion as three main measurement dimensions of visual esthetics. Finally, we took the destination choice intention of tourists as the behavioral R.

## Hypothesis Development

### Visual Appeal

Many disciplines have considered that visual appeal is essential in the past decades, directly affecting esthetic perception. Bloch (1995) suggested that good product design will attract consumers to buy, communicate with them, and increase the value of a product by improving the quality of the user experience associated with it. Research on visual appeal and website design shows that it has an immediate effect on the esthetic perception of users, which leads to the formation of their first impression of the website, which forms judgment of the product (Lindgaard et al., 2006). In addition, many scholars believed that visual appeal has a direct impact on consumer behavior intention, attention, and participation (Pengnate et al., 2019).

In tourism, esthetic evaluation can be considered from the perspective of visual appeal to a large extent. Visual appeal is considered to be visual esthetics, natural beauty, or attraction in this field. In the early study of visual esthetics, color, texture, terrain, and other landscapes were considered (Kirillova et al., 2014). According to Kim and Moon (2009), the impact of visual attractiveness on tourists was not only in the natural environment but also in restaurants or hotels. Many scholars later confirmed that the visual appeal of the hotel was somehow more important than the hotel facilities. The interior design of hotels, such as color, lighting, decorating style, and furnishings, will attract customers to visit and affect customer experience and evaluation (Kirillova and Chan, 2018). These studies have revealed that tourists are attracted by the physical beauty of the destination and emphasize the importance of visual appeal. There is no doubt about the role of visual appeal in destinations. Tourists rely on media representation of their destinations to make pre-tourism decisions, such as websites and tourism photos (Zhang et al., 2018). In previous advertising and marketing research, it has been confirmed that high-quality and professional esthetic photos can stimulate consumer responses (Lohse and Rosen, 2001). Moreover, professional visual esthetic is more attractive to tourists than amateurish (Marder et al., 2021). Therefore, tourism advertising also pays great attention to the photos or the visual effects of esthetics. Use these to present the best image of the destination to inspire visitors to travel (Litvin and Mouri, 2009). Therefore, based on the above analysis, the hypotheses of this study are as follows:

**Hypothesis 1**: There are significant differences in the visual appeal of tourists when watching tourism images with different environmental esthetic types. Tourists will perceive tourism images with the natural environment (vs. built environment) to be more (vs. less) visually appealing.

**Hypothesis 2:** There are significant differences in the visual appeal of tourists when watching tourism images with different photograph esthetic types. Tourists will perceive tourism images with professional esthetics (vs. amateur esthetics) to be more (vs. less) visually appealing.

**Hypothesis 3:** The visually appealing has a positive effect on the choice intention of tourists.

### First Impression

A first impression is a mental activity that is formed and recorded in a short period of time (Kim, 2019). The first impression emerges immediately during the first encounter with a subject. Once the first impression has been formed, it is preserved for a long time, even if there is no further contact with the stimulus, and also affects subsequent evaluations (Beerli and Martín, 2004). This long-term effect of a first impression can be interpreted as a phenomenon called the “halo effect,” which means that global evaluations influence the evaluations of individual attributes (Nisbett and Wilson, 1977). Lindgaard et al. (2006) showed that people can identify visual esthetic differences and form a first impression within 50 m.

For example, on the one hand, many scholars have regarded the esthetic features of a website (such as visual complexity, page order, and novelty) as the antecedent variable of a first impression and have explored the influence of these esthetic features on the first impressions of people. Then, these scholars have studied the first impression of the website of users, which was found to affect assessments of the credibility, attractiveness, participation, and intention to use the website (Kim, 2019). On the other hand, some researchers have suggested that visual elements (e.g., character images, brand images, and product images) used on a hotel website can help improve the first impression and thus, increase sales (Xia et al., 2020). In the tourism literature, participants can make fast judgments about travel sites, and inspiration and usability are the main drivers of a good first impression, which can increase travel planning (Xiang and Fesenmaier, 2005). Levene (2010) showed that when a potential tourist evaluates the relevance and usefulness of a website for the first time, it only takes a short time for them to evaluate the website and form an overall impression. In other words, a rapid and almost unconscious (but complex) thought process is activated when the travel planner visits the website. Such a reaction is instantaneous but rational, and the brain tries to categorize and filter a website into a certain type (e.g., maybe approve, maybe disapprove, or uncertain). Therefore, immediate interaction with a webpage evokes the first impression, which is more likely to enable users to make a rapid choice about a website and even subsequent decisions (Kim and Fesenmaier, 2008). In addition, Ieiri et al. (2017) studied first impressions about tourist attractions and found that a good first impression helps increase the satisfaction and motivation of tourists to revisit. Therefore, based on the above analysis, the hypotheses of this study are as follows:

**Hypothesis 4:** There are significant differences in the first impression of tourists when watching tourism images with different environmental esthetic types. Tourists will perceive tourism images with the natural environment (vs. built environment) to be better (vs. worse) first impressions.

**Hypothesis 5:** There are significant differences in the first impression of tourists when watching tourism images with different photograph esthetic types. Tourists will perceive tourism images with professional esthetics (vs. amateur esthetics) to be better (vs. worse) first impressions.

**Hypothesis 6:** The first impression has a direct and positive influence on the choice intention of tourists.

### Esthetic Emotions

Emotions can be described as a relatively transient physiological, empirical, and behavioral response to motivational internal and external stimuli (Gross and Thompson, 2007). Arousal is defined as “the degree to which an individual feels excited, stimulated, alert, or activity.” Pleasure is the hedonic price (pleasurable or unpleasant) of an emotional response to a stimulus that enables the consumer to achieve its important goals (Moyle et al., 2019). Emotion is becoming a core idea in tourism research. The demand of people for outings can be affected by emotions. Thus, the emotion toward the destination may encourage tourists to plan a trip. Previous research suggests that the negative and positive emotions of tourists can regulate the emotional intensity and affect their attitudes, satisfaction, and behavior (Hosany and Gilbert, 2010). Besides, many scholars have begun to fix their eyes on tourism advertisements, promotional materials, web design, etc. This can stimulate the emotions and motivations of tourists and affect the emotional reactions and behavioral intentions of tourists (Li et al., 2018).

Esthetic emotions are traditionally the emotions that can arise when an individual perceives and evaluates the esthetic appeal or virtues of a stimulus. The esthetic processing can stimulate the brain, elicit individual positive emotions, and make a person experience a sense of pleasure and satisfaction (Li et al., 2018). In tourism, esthetic emotions can trigger the motivation of tourists and influence their destination choice, revisit, and recommendation to others (Schindler et al., 2017). In the field of hotel research, a large number of studies have found that visual esthetical designs are passionate by managers. That is because visual esthetics usually been seen as an important aspect of the perceived value of consumers, and managers would like to influence the esthetic emotions of consumers through esthetic design and influence their behavioral intentions further (Breiby and Slatten, 2015). Therefore, based on the above analysis, the hypotheses of this study are as follows:

**Hypothesis 7:** There are significant differences in the esthetic emotions of tourists when watching tourism images with different environmental esthetic types. Tourists will perceive tourism images with the natural environment (vs. built environment) to be more (vs. less) positive esthetic emotions.

**Hypothesis 8:** There are significant differences in the esthetic emotions of tourists when watching tourism images with different photograph esthetic types. Tourists will perceive tourism images with professional esthetics (vs. amateur esthetics) to be more (vs. less) positive esthetic emotions.

**Hypothesis 9:** The positive esthetic emotions significantly affect on the choice intention of tourists.

## Materials and Methods

### Eye-Tracking

Eye-tracking is a relatively new technique for studying visual attention and perception in tourism research. Based on the hypothesis that eye movement indicates the focus of the attention of a person, many studies have shown that eye-tracking can more objectively evaluate visual effects than self-reports (Scott et al., 2016). Eye-tracking can capture objective and real-time data of visual attention, and it is also an indicator of good behavior in information acquisition, which is considered to be closely related to higher-order cognitive processes (Baek and Ok, 2017). Commonly used eye-tracking measurement indicators are fixation duration, fixation count, pupil size, etc., which reveal different aspects of visual esthetic and esthetic emotion (Zhang et al., 2021).

### Participants and Preparation

Notably, 64 people participated in this study, of which 28 (43.75%) were women. Their ages ranged from 20 to 30 years. Participants were recruited from students in Fuzhou. All participants had a full-color vision and normal or corrected-to-normal vision. Participants signed an informed consent form and confirmed the eye-tracking experiment procedure. This study complied with departmental ethics committee regulations.

Images were sourced from the National 5A Tourist Area that is used by tourism businesses across China in their marketing activities. Concretely speaking, four natural environmental tourist attractions and four built environmental landscapes were selected from the National 5A Tourist Area. Each landscape selects three different popular sights, and each sight comprises one photo taken by tourist publicity material (as professional esthetics) and one photo from online platforms taken by tourists (as amateur esthetics). Notably, 48 experimental images were processed for color and brightness. We designed a 2 (environmental esthetics: natural and built) (within-subjects) × 2 (photograph esthetics: professional and amateur) (between-subjects) mixed-design. Predictive tests confirmed that experimental images taken by the tourist publicity material were esthetically perceived to be more professional than their amateur counterparts (*M*_*Professional*_ = 6.17, *SD* = 0.23, vs. *M*_*Amateur*_ = 4.16, *SD* = 0.31, *P* < 0.001) using a 7-point scale (1 = very amateur to 7 = very professional). The instrument used in the experiment was a Hi-Speed Eye Tracking System produced by the SMI Company. Based on the infrared light to create reflection patterns on the eye corneas of participants, a 500 Hz sampling frequency was used. The Experiment Center software [SensoMotoric Instruments (SMI), Brandenburg, Germany] was used to design the experiment and to play the stimulus material. Completed eye calibration and eye-tracking data were recorded using iViewX3.5 software [SensoMotoric Instruments (SMI), Brandenburg, Germany]. Finally, BeGaze 3.5 software [SensoMotoric Instruments (SMI), Brandenburg, Germany] was used to complete the extraction and analysis of eye-tracking data.

### Procedure

The experimental process of this study consists of two parts. First, various eye-tracking data were recorded and analyzed using an eye-tracking instrument. Following the eye-tracking task, participants evaluated the images through a questionnaire about the emotional, first impression, visual appeal, and choice intention. SPSS26.0 [International Business Machines Corporation (IBM), New York, United States] was used for the ANOVA.

**FIGURE 2:**
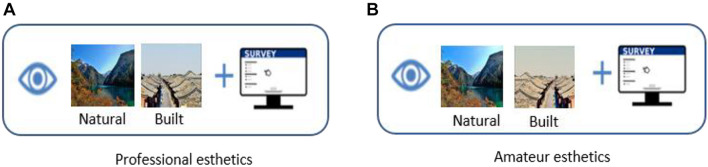
Procedure for eye-tracking research.

All participants were randomly assigned to professional or amateur conditions. The images were presented randomly, and each image was displayed for 10 s, and subjects were asked to press a button “Yes” or “No” as a choice based on their willingness to visit the destination. The experiment was divided into four parts, and each part can rest for 2 min. After the eye-tracking task, the computer presented 24 images again. Then, each image was presented with questions asking participants to rate the visual appeal (e.g., this environment image is very attractive), emotion (e.g., this environment image makes me very excited), first impression (e.g., to which extent do you think the first impression about this photo affect the assessment of its?), and choice intention (e.g., would you choose this environment image to go). The measurement of visual appeal was suggested by Cyr et al. (2006), the emotion measurement by Mano and Oliver (1993), the measurement of the first impression by Becerra (2016), and the choice intention measurement by Walters et al. (2012). Participants were asked to score each item using a 7-point scale. After the whole experiment is completed, some reward is given to the participants. The procedure for eye-tracking research is shown in Figure 2.

## Data Analysis and Results

For each dependent variable of average fixation duration, first fixation duration, average fixation count, and average pupil size, we conducted a 2 (environmental esthetics) × 2 (photograph esthetics) ANOVA. Then, we analyzed the visual appeal, arousal, and choice intention of self-reported images in the same manner, as shown in Table 1.

**TABLE 1:** Descriptive statistics.

**Item**	**Natural-professional**	**Natural-amateur**	**Built-professional**	**Built-amateur**
Average fixation time	8.24 (0.85)	6.50 (1.01)	7.01 (0.90)	6.04 (0.56)
Average time for first fixation	0.33 (0.05)	0.30 (0.04)	0.29 (0.03)	0.27 (0.04)
Average fixation count	24.99 (2.95)	19.90 (3.11)	21.60 (1.81)	16.80 (2.10)
Average pupil size	5.37 (0.23)	4.63 (0.22)	4.97 (0.23)	4.42 (0.22)
Visual appeal	5.65 (0.33)	5.36 (0.48)	5.27 (0.55)	4.88 (0.43)
Esthetics emotion	5.99 (0.50)	5.72 (0.41)	5.66 (0.54)	4.95 (0.70)
First impression	6.11 (0.55)	5.93 (0.25)	5.61 (0.59)	5.55 (0.43)
Choice intention	6.35 (0.65)	6.03 (0.53)	5.24 (0.44)	4.83 (0.73)

### Eye-Tracking Data Analysis

Cui et al. (2020) proposed that the fixation counts, fixation time, and pupil diameter are used as a measure of visual appeal. As for the H1, the higher visually appealing, the greater fixation time [*M*_*natural*_ = 7.14, *SD* = 0.93, *M*_*built*_ = 6.52, *SD* = 0.73, *F*(1,62) = 28.956, *P* < 0.001] and fixation count [*M*_*natural*_ = 22.44, *SD* = 3.03, *M*_*built*_ = 19.20, *SD* = 1.95, *F*(1,62) = 46.771, *P* < 0.001], the larger pupil size [*M*_*natural*_ = 5.01, *SD* = 0.22, *M*_*built*_ = 4.70, *SD* = 0.22, *F*(1,62) = 53.696, *P* < 0.001] on environmental esthetics. Thus, H1 was supported. Similarly, H2 was confirmed in that the higher visually appealing, the greater fixation time [*M*_*Professional*_ = 7.62, *SD* = 0.87, *M*_*Amateur*_ = 6.27, *SD* = 0.78, *F*(1,62) = 74.263, *P* < 0.001], fixation count [*M*_*Professional*_ = 23.29, *SD* = 2.38, *M*_*Amateur*_ = 18.35, *SD* = 2.60, *F*(1,62) = 108.787, *P* < 0.001], larger pupil size [*M*_*Professional*_ = 5.17, *SD* = 0.23, *M*_*Amateur*_ = 4.53, *SD* = 0.22, *F*(1,62) = 235.487, *P* < 0.001] on photograph esthetics, as shown in Tables 1, 2.

**TABLE 2:** ANOVA results: eye-tracking data.

**Item**	**Dependent variable**	***F***	**Significance**
Environmental esthetics	Average Fixation Time	28.956***	0.000
	Average Fixation Count	46.771***	0.000
	Average Time for First Fixation	4.765*	0.05
	Average Pupil Size	53.696***	0.000
Photographic esthetics	Average Fixation Time	74.263***	0.000
	Average Fixation Count	108.787***	0.000
	Average Time for First Fixation	4.233*	0.05
	Average Pupil Size	235.487***	0.000
Environmental esthetics *	Average Fixation Time	5.787*	0.018
	Average Fixation Count	0.092	0.763
Photographic esthetics	Average Time for First Fixation	1.34	0.260
	Average Pupil Size	5.044*	0.027

***P < 0.001; **P < 0.01; *P < 0.05.

Sheng et al. (2013) found that when user formed their first impressions, the total fixation time was greater for websites that received normally good impressions than for websites that obtained normally unfavorable impressions. Besides, the time for first fixation can be selected for a key indicator to build first impressions of users as suggested by Li et al. (2015). Regarding the H4, the better first impressions, the greater fixation time [*M*_*natural*_ = 7.14, *SD* = 0.93, *M*_*built*_ = 6.52, *SD* = 0.73, *F*(1,62) = 28.956, *P* < 0.001] and the time for first fixation [*M*_*natural*_ = 0.31, *SD* = 0.05, *M*_*built*_ = 0.28, *SD* = 0.04, *F*(1,62) = 4.765, *P* < 0.05] on environmental esthetics. Thus, H4 was supported. Similarly, H5 was confirmed in that the higher visually appealing, the greater fixation time [*M*_*Professional*_ = 7.62, *SD* = 0.87, *M*_*Amateur*_ = 6.27, *SD* = 0.78, *F*(1,62) = 74.263, *P* < 0.001] and the time for first fixation [*M*_*Professional*_ = 0.31, *SD* = 0.04, *M*_*Amateur*_ = 0.28, *SD* = 0.04, *F*(1,62) = 4.233, *P* < 0.05] on photograph esthetics, as shown in Tables 1, 2.

Another study showed that the pupil diameter indicator response can reflect esthetic emotions of an individual, especially when viewing positive esthetic emotions (Ho and Lu, 2014). For H7, the more positive esthetic emotions, the larger pupil diameter [*M*_*natural*_ = 5.01, *SD* = 0.22, *M*_*built*_ 4.70, *SD* = 0.22, *F*(1,62) = 53.696, *P* < 0.001] on environmental esthetics. Similarly, H8 was confirmed in that the more positive esthetic emotions, the larger pupil size [*M*_*Professional*_ = 5.17, *SD* = 0.23, *M*_*Amateur*_ = 4.53, *SD* = 0.22, *F*(1,62) = 235.487, *P* < 0.001] on photograph esthetics, as shown in Tables 1, 2.

In addition, the interaction effects between the two independent variables on the eye-tracking data were examined by MANOVA. With regard to environmental esthetics, the photograph esthetics did not affect fixation count of viewers [*F*(1,62) = 0.092, *P* > 0.05] and the time for first fixation [*F*(1,62) = 1.34, *P* > 0.05]. However, with regard to environmental esthetics, the photograph esthetics affect fixation time of viewers [*F*(1,62) = 5.787, *P* < 0.05] and pupil size [*F*(1,62) = 5.044, *P* < 0.05].

### Self-Report Data Analysis

ANOVA of 2 (environmental esthetics) × 2 (photograph esthetics) for visual appeal found significant effects for photograph esthetics [*F*(1,62) = 23.380, *P* < 0.001]; the main effect of environmental esthetics was significant [*F*(1,62) = 29.268, *P* < 0.001]; in addition, we found interaction effect for environmental esthetics and photograph esthetics [*F*(1,62) = 5.044, *P* < 0.05].

ANOVA of 2 (environmental esthetics) × 2 (photograph esthetics) for first impressions found no significant effects for photograph esthetics [*F*(1,62) = 0.452, *P* > 0.05]; the main effect of environmental esthetics was significant [*F*(1,62) = 15.62, *P* < 0.001]; in addition, we found no interaction effect for environmental esthetics and photograph esthetics [*F*(1,62) = 2.085, *P* > 0.05].

ANOVA of 2 (environmental esthetics) × 2 (photograph esthetics) for esthetic emotions found significant effects for photograph esthetics [*F*(1,62) = 16.178, *P* < 0.001]; the main effect of environmental esthetics was significant [*F*(1,62) = 26.353, *P* < 0.001]. However, there was no interaction effect for environmental esthetics and photograph esthetics [*F*(1,62) = 0.294, *P* > 0.05], as shown in Tables 1, 3.

**TABLE 3:** ANOVA results: self-report data.

**Item**	**Dependent variable**	***F***	**Significance**
Photographic esthetics	Visual Appeal	23.380***	0.000
	First impressions	0.452	0.771
	Esthetic emotions	16.178***	0.000
Environmental esthetics	Visual Appeal	29.268**	0.000
	First impressions	15.62**	0.000
	Esthetic emotions	26.353***	0.000
Photographic esthetics *	Visual Appeal	4.920*	0.028
Environmental esthetics	First impressions	2.085	0.092
	Esthetic emotions	0.294	0.589

***P < 0.001; **P < 0.01; *P < 0.05.

From the results of the self-report data, it can be found that there were significant differences in visual appeal and esthetic emotions among different photograph esthetics and environmental esthetics. Specifically, the potential tourists viewing natural environment images have more positive esthetic emotions than they watch built environment images. The potential tourists have more positive esthetic emotions by viewing professional esthetic images rather than watching amateur esthetic images. However, there were no significant differences in first impressions with different photograph esthetics, and the main effect of environmental esthetics was significant in first impressions. Many studies have shown that eye-tracking can more objectively evaluate visual effects than self-reports (Scott et al., 2016). Eye-tracking can capture objective and real-time data of visual attention, and it is also an indicator of good behavior in information acquisition, which is considered to be closely related to higher-order cognitive processes (Baek and Ok, 2017). Therefore, we considered that there were significant differences in first impressions with different photograph esthetics by considering the eye-tracking data.

### Gaze Plot

**FIGURE 3:**
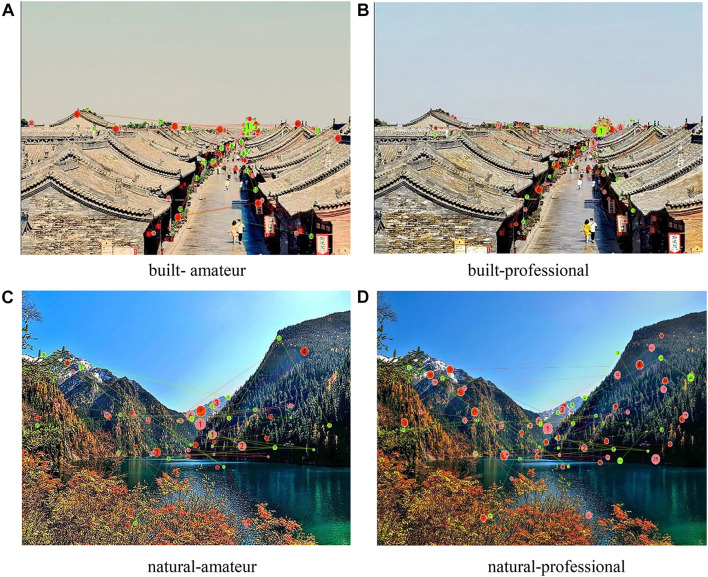
A scan path showing fixation (circles) and saccades (lines) (*n* = 3). **(A)** Built-amateur; **(B)** Built-professional; **(C)** Natural-amateur; and **(D)** Natural-professional.

**FIGURE 4:**
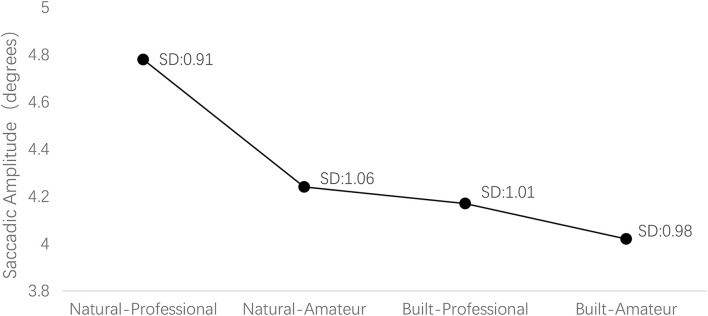
Saccadic length.

Previous studies have outlined that the scan path was longer on hedonic contents than on neutral content (Kuzinas et al., 2016). In addition, viewing complex scenes prompted shorter scan paths (Brunyé and Gardony, 2017). In other words, scan paths tend to increase when visual search task becomes more simple or hedonic. Figures 3, 4 illustrate that the mean saccadic length while watching the natural environment images was significantly longer than watching the built environment images and viewing professional esthetics prompted longer mean saccadic length than amateur esthetic images. Therefore, this indicated that the visual search task becomes more difficult when viewing built environment or amateur esthetic content, and visual search task becomes more hedonic when viewing natural environment images or professional esthetic images.

### Correlation Analysis and Regression Analysis

A correlation analysis shows that, as shown in Table 4, a high correlation exists between visual appeal and average fixation time, esthetic emotion, first impression, average time for the first fixation, average pupil size, and average fixation count. In addition, esthetic emotion was associated with the first impression, average fixation time, average time for first fixation, average fixation count, and average pupil size. In addition, the first impression was associated with the average time for first fixation, average time for first fixation, average pupil size, and average fixation count. From this correlation analysis, it can be concluded that the visual appeal first impression and esthetic emotion in the self-report are related to the average fixation time, average time for first fixation, average fixation count, and average pupil size of the eye-tracking data. Therefore, in this study, eye-tracking data can be used as strong objective evidence to judge whether an image has a visual appeal first impression and esthetic emotion.

**TABLE 4:** A correlation analysis.

	**Visual appeal**	**Esthetic emotion**	**First impression**	**Average fixation time**	**Average time for first fixation**	**Average fixation count**	**Average pupil size**
Visual appeal	1						
Esthetic emotion	0.250**	1					
First impression	0.590**	0.378**	1				
Average fixation time	0.309**	0.423**	0.298**	1			
Average time for first fixation	0.122*	0.187*	0.360**	0.333**	1		
Average fixation count	0.251**	0.354**	0.166*	0.520**	0.256**	1	
Average pupil size	0.504**	0.363**	0.551**	0.525**	0.109*	0.438**	1

**P < 0.01; *P < 0.05.

The value of skewness was from 0.071 to 0.122, and kurtosis was from −0.544 to −0.792, which can conform to the normal distribution. Visual appeal, esthetic emotion, and first impression were used as independent variables, and the choice intention was used as dependent variables for multivariate linear regression, as shown in Table 5. Thus, we concluded that visual appeal, esthetic emotion, and first impression have a positive effect on the choice intention of tourists. The hypotheses H3, H6, and H9 are supported.

**TABLE 5:** Multiple linear regression.

	**Predictor**	***B***	***S.E.***	**Beta**	***t***	**Sig**	**VIF**
Model1 (Influence of visual appeal and arousal on choice intention)	Intercept	−0.663*	0.609		−1.986	0.045	
	Esthetic emotion	0.261 *	0.101	0.163	2.581	0.011	1.067
	Visual appeal	0.877***	0.080	0.691	10.908	0.000	1.067
	First impression	0.575**	0.038	0.263	3.01	0.002	1.067
	*R* ^2^	0.560***					
	Adjusted *R*^2^	0.553***					

*^*a*^Dependent variable: choice intention.*

*^*b*^Independent variable: visual appeal, esthetic emotion, first impression.*

***P < 0.001; *P < 0.05.

## Conclusion and Discussion

Few scholars use destination images as visual perceptible materials to study the influence of destination choice intention from the perspective of visual esthetics. To fill the gap, this study has explored the connection of visual esthetics to the destination choice intention of tourists. Thus, we sought to use an S-O-R paradigm to predict destination choice intention, which includes three variables related to visual esthetics as follows: first impression, visual appeal, and esthetic emotion. We used the combination of self-reported and eye movement data to examine the cognitive processes of tourists for visual esthetic formation.

According to the results of the self-reported and eye movement data analysis, nine hypotheses were supported. Compared to the built environment and amateur esthetic images, the natural environment and professional esthetic images got a higher visual appeal. Hadinejad et al. (2019) found that eye-tracking data can be used as a reliable indicator to perceive the beauty of natural scenery. It can compare and identify the most beautiful and attractive images in natural scenery. At present, with research toward esthetics, they have been evaluated based mainly on complexity and symmetry. Our research, esthetics based on a visually appealing evaluation, demonstrates that the esthetic brings a pleasing experience. The natural environment is more visually appealing and has a greater impact on the pleasing experience of people. In addition, professional esthetics are esthetically pleasing content and should yield more positive responses. Professional photography pays more attention to factors such as symmetry and clarity, which are the key to visual appeal (Marder et al., 2021). Previous study in this area has mainly focused on the photographer themselves and their motivations. But their amateur esthetic photos whether they can affect the perception and motivation of tourists is less proof. Meanwhile, many studies lack an objective way to evaluate visual effects. This study fills this gap, and many indicators were evaluated for professional esthetic images by using eye-tracking.

The eye-tracking results indicate that participants spend more first fixation time and fixation time on the natural environment and professional esthetic photographs than built environment and amateur esthetic photographs. The fixation time was greater for the photograph that received favorable impressions than for the photograph that received unfavorable impressions (Sheng et al., 2013). The visual appeal is usually positively related to first impressions. As mentioned in the above analysis of visual appeal, we can assume that objects with high visual appeal usually can form positive first impressions. Besides, previous study explored the influence of the esthetic features of a website (such as visual complexity, page order, and novelty) on first impressions of people (Kim, 2019). In esthetic features, natural environment and professional esthetic photographs are more attractive than built environment and amateur esthetic photographs. Therefore, we speculated that the natural environment and professional esthetic photographs have a better first impression.

Pupillary responses can record esthetic emotions. The higher pupil dilation is attributed to positive esthetic emotions and contents with high processing fluency stimuli (Kuchinke et al., 2009). Meanwhile, the scan path was associated with pleasurable content and complex cognitive processing (Brunyé and Gardony, 2017). We found more fixation counts, larger pupil size, as well as greater saccade lengths when individuals viewed the natural environment and professional esthetic images. So, we think that natural environment and professional esthetic images have higher visual processing fluency (or less cognitive effort) and positive esthetic emotions than built environment and amateur esthetic images. Previous studies have suggested that the fixation count and the eye movement distance would be greater in viewing built images compared with nature images. Mainly, since compared with processing nature images, processing built images require greater cognitive effort (Franěk et al., 2018). On the contrary, our study suggests that greater cognitive effort results in less fixation counts and shorter scan paths. It can be mainly explained as follows: first, being close to nature provides a series of psychological benefits. A natural scene can arouse positive emotions, which indicates visual processing is more fluent, and less cognitive effort is required. Second, by watching pictures with hedonic contents, the number of fixation counts was higher than others. In addition, emotional arousal has been found to increase fixation counts (Kuzinas et al., 2016). Third, professional esthetic images with high quality have been found to stimulate optimal consumer responses and may lead to a positive spillover. However, amateur esthetic appears to include some “messy,” generally lacking beauty (Marder et al., 2021).

To theoretically examine how visual esthetic affects destination choice intention. We constructed a visual esthetic model, which has predominantly examined visual appeal, first impression, and esthetic emotion in impacting destination choice intention derived from environment esthetics and photograph esthetics. The relationship between tourist behavior and emotion is strong and direct (Le et al., 2020). Visual appeal is important for choice behavior (Jantathai et al., 2013). A good first impression helps increase the satisfaction and motivation of tourists to revisit (Ieiri et al., 2017). Previous studies have seldom considered these three constructs together to predict destination choice intention within the realm of tourism marketing. Based on the S-O-R model, this study integrates these visual esthetic variables into a novel conceptual framework to deepen understanding the destination choice intention, which fills the research gap in the destination choice literature.

From the academic perspective, this study makes the following contributions. First, this study is to deepen the understanding of tourism photography by developing and testing a theoretical model that accounts for the relationships between visual esthetics and destination choice intention of tourists. Second, this study explores the effects of first impression, visual appeal, and emotion on destination choice intention by visual esthetic perspective. Third, reliable eye-tracking indicators were proposed to evaluate visual esthetics.

Our studies provide practical guidance value for destination managers. This study can help travel destination managers to better understand the role of photographic esthetics and understand the relationship between visual features and the visual appeal, first impression, and emotional arousal of potential tourists. Tourism managers should invest in more appealing and emotional photos to be used for social media to drive tourists to visit. If the online website policy promises, we suggest tourism providers can consider giving priority to professional pictures to potential tourists. That can create a better first impression of the tourist destination. Meanwhile, destination managers can give tourists some economic incentive to encourage them to take high-quality photos. Moreover, destination staff can embellish the photos to make tourists feel relaxed, arousing, and finally attract tourists. For the built environment scenic, they can add some natural elements to the layout, which may increase the arousal of tourists and stimulate the visit intention of tourists. For example, hotels can attract tourists in an architectural style and append the natural environment layout, to attract more tourists.

## Limitations and Future Work

This study has a few limitations. First, the small sample of eye-tracking is one of the limitations of this study. This is mainly limited by experimental conditions and cost. Second, visual esthetics involves esthetic judgment, which is not discussed in our study. Regarding future research, we may consider more types and try to expand more samples. Future research can pay attention to whether discuss the first impression, visual appeal, and esthetic emotion that differs with different thinking styles, which has not been mentioned in this study. Tourists can make travel decisions based on their logic-driven dispositions or based on emotion-driven dispositions. Therefore, tourists with different modes of thinking have great differences in decision-making.

## Data Availability Statement

The raw data supporting the conclusions of this article will be made available by the authors, without undue reservation.

## Ethics Statement

The studies involving human participants were reviewed and approved by the Ethics Committee of School of Humanities and Social Sciences at Fuzhou University. The patients/participants provided their written informed consent to participate in this study. Written informed consent was obtained from the individual(s) for the publication of any potentially identifiable images or data included in this article.

## Author Contributions

YL and WD: conceptualization. LC: methodology, resources, writing—review and editing, project administration, and funding acquisition. WD: software, formal analysis, investigation, data curation, writing—original draft preparation, and visualization. YL: supervision. LC, YL, and WD: validation. All authors have read and agreed to the published version of the manuscript.

## Conflict of Interest

The authors declare that the research was conducted in the absence of any commercial or financial relationships that could be construed as a potential conflict of interest.

## Publisher’s Note

All claims expressed in this article are solely those of the authors and do not necessarily represent those of their affiliated organizations, or those of the publisher, the editors and the reviewers. Any product that may be evaluated in this article, or claim that may be made by its manufacturer, is not guaranteed or endorsed by the publisher.
